# MiR-146a rs2910164 G/C polymorphism and gastric cancer susceptibility: a meta-analysis

**DOI:** 10.1186/s12881-014-0117-2

**Published:** 2014-10-20

**Authors:** Zhong Xu, Lingling Zhang, Hui Cao, Banjun Bai

**Affiliations:** Department of Gastroenterology, Guizhou Provincial People’s Hospital, The Affiliated People’s Hospital of Guiyang Medical University, Guiyang, 550002 Guizhou Province PR China; Department of Oncology, Guizhou Provincial People’s Hospital, The Affiliated People’s Hospital of Guiyang Medical University, Guiyang, 550002 Guizhou Province PR of China

**Keywords:** microRNA-146a, Stomach neoplasms, Polymorphism, Meta-analysis

## Abstract

**Background:**

Evidence has shown that single nucleotide polymorphism located in pre-miRNA or mature microRNA may modify various biological processes and affect the processing of carcinogenesis. Published results about the association between miR-146a rs2910164 G/C polymorphism and human gastric cancer susceptibility are inconclusive. The aim of this study was to acquire a more precise effect of the association between the miR-146a rs2910164 polymorphism and gastric risk by meta-analysis.

**Methods:**

Eligible genetic association studies were searched from PubMed, Web of Knowledge and Chinese Biomedicine Database on human subject. Quantitative data synthesis was conducted for the associations of miR-146a rs2910164 G/C polymorphism with susceptibility to gastric cancer.

**Results:**

Nine eligible studies that included a total of 3,885 gastric cancer patients and 5,396 controls were identified in the present meta-analysis. The overall OR indicated a potential association between rs2910164 polymorphism and GC but the effect was not statistically significant (GG vs. CG/CC: OR = 1.076, 95% CI 0.925-1.251, P = 0.342). When stratifying for population, the result showed that miR-146a rs2910164 GG genotype was associated with increased gastric cancer risk among Chinese in recessive model (GG vs. CG/CC: OR = 1.171, 95% CI 1.050-1.306, P = 0.005). Besides, no significant difference was found in gender, smoking, location, metastasis of lymph node and Laurèn’s classification.

**Conclusions:**

The present meta-analysis suggests an increased risk between miR-146a rs2910164 GG genotype and gastric cancer susceptibility in Chinese based on published literatures.

## Background

Gastric cancer (GC) is still one of the most common causes of cancer-related death worldwide despite an overall decrease in incidence over the past 10 years [1,2]. It is generally accepted that individual genetic susceptibility has an important role in the pathogenesis of tumor including GC.

MicroRNAs (miRNAs/miR) are endogenous 18–24 nucleotide noncoding RNAs that could regulate gene expression and sequentially regulate various biological processes [3,4]. Functional characterization of some microRNAs in cancer initiation and progression indicates that they might play a more important role in the pathogenesis of human cancers [5,6]. The relationship between miRNA and cancer has been extensively studied since 2002, the first demonstration of a link between miRNA genes and cancer [7].

Studies have shown that the polymorphisms in pre-miRNA or mature microRNA may modify various biological processes by influencing the expression and/or target selection of microRNAs [8,9]. Over the past decade, numerous studies identified genetic variants in the precursor or mature miRNA sequence of miR-146a rs2910164 (G > C), which have been reported to be associated with breast cancer and hepatocellular carcinoma [10,11].

A number of studies were published to describe the association between miR-146a rs2910164 G/C polymorphism and cancer risk in recent years. A meta-analysis reported by Lian et al. [10] concluded that the CC homozygote of rs2910164 may contribute to breast cancer susceptibility among Europeans. Another meta-analysis demonstrated that there is no significant correlation between has-miR-146a rs2910164 polymorphism and breast cancer risk [12]. A HuGE meta-analysis in 2013 failed to find any significant association between rs2910164 polymorphism and the risk of breast cancer [13]. Based on systematic review, Chen et al. [14] reported that quality of evidence was low for rs2910164 SNP genetic association with lung cancer. Several meta-analyses pooled the association between rs2910164 polymorphism and cancer risk in general [15,16]. Some meta-analysis reported the rs2910164 polymorphism and gastrointestinal cancer susceptibility [17-19].

However, published data on the association between miR-146a rs2910164 G/C polymorphism and human gastric cancer susceptibility are inconsistent and inconclusive. Therefore, we performed the present meta-analysis to quantitatively estimate the association of miR-146a G/C SNP with gastric cancer risks.

## Methods

### Literature search strategy

Electronic databases including PubMed, Web of Knowledge and Chinese Biomedicine Database (CBM) were searched for relevant studies conducted on human subject until July 2014. The following search keywords were used: “gastric” or “stomach”, “neoplasm” OR “carcinoma” OR “cancer” OR “tumor” OR “adenocarcinoma” and “miR-146a OR “miRNA-146a” OR “microRNA-146a” OR “has-miR-146a”. To further identify potentially relevant studies, the reference lists of articles identified in the initial search were also scanned manually. We directly contacted authors for related data that were unavailable in the original publications.

### Inclusion and exclusion criteria

Studies were considered eligible if they met all of the following criteria: (1) explore the association between miR-146a polymorphism and human GC risk, (2) design as case–control studies, (3) identification of gastric cancer cases was confirmed histologically or pathologically. Studies were excluded based on any of the following reasons: (1) no sufficient data reported, (2) articles of letters, reviews, case reports, conference abstracts, editorials or expert opinion. If serial studies of the same population from the same research group were published, only the largest series were selected.

### Data extraction

The final set of articles was assessed independently by two reviewers (Zhong Xu and Lingling Zhang) according to the inclusion criteria listed above. Disagreements were resolved by discussion. The following information was retrieved from each report: first author, publication year, study population, ethnicity, number of GC cases and controls, frequency of genotypes, and genotyping method.

### Statistical analysis

Hardy–Weinberg equilibrium (HWE) status was examined by Pearson’s goodness-of-fit χ^2^ test in each study (*P* <0.05 was considered as significant disequilibrium in the control group). The strength of the association between GC and the miR-146a rs2910164 polymorphism was assessed by calculating odds ratio (OR) with 95% confidence interval (CI). The pooled ORs were obtained from combination of single studies by homozygote comparison (GG vs. CC), heterozygote comparison (CG vs. CC), dominant and recessive models (CG/GG vs. CC, and GG vs. CG/CC), allele comparison (G vs. C), respectively. The analysis of heterogeneity between studies was determined by using the Cochrane’s Q test and *I*^2^-test (*P* <0.10 or *I*^2^ > 50% was considered indicative of statistically heterogeneity) [20]. When there was no significant heterogeneity existing among studies, the pooled ORs estimate of each study was calculated by the fixed-effects model. Otherwise, the random-effects model was employed. Subgroup analyses were stratified by the study characteristic if there were enough reports. Sensitivity analysis was carried out by deleting one single study each time to examine the influence of individual data set on the pooled ORs. Publication bias was assessed by using the methods of Begg’s funnel plots and Egger’s test (*P* <0.05 was considered representative of statistically significant publication bias) [21,22]. Statistical analyses performed by using the software Stata12.0 (Stata Corpotion, College Station, Texas).

## Results

### Studies selection and characteristics of the included studies

**Figure 1:**
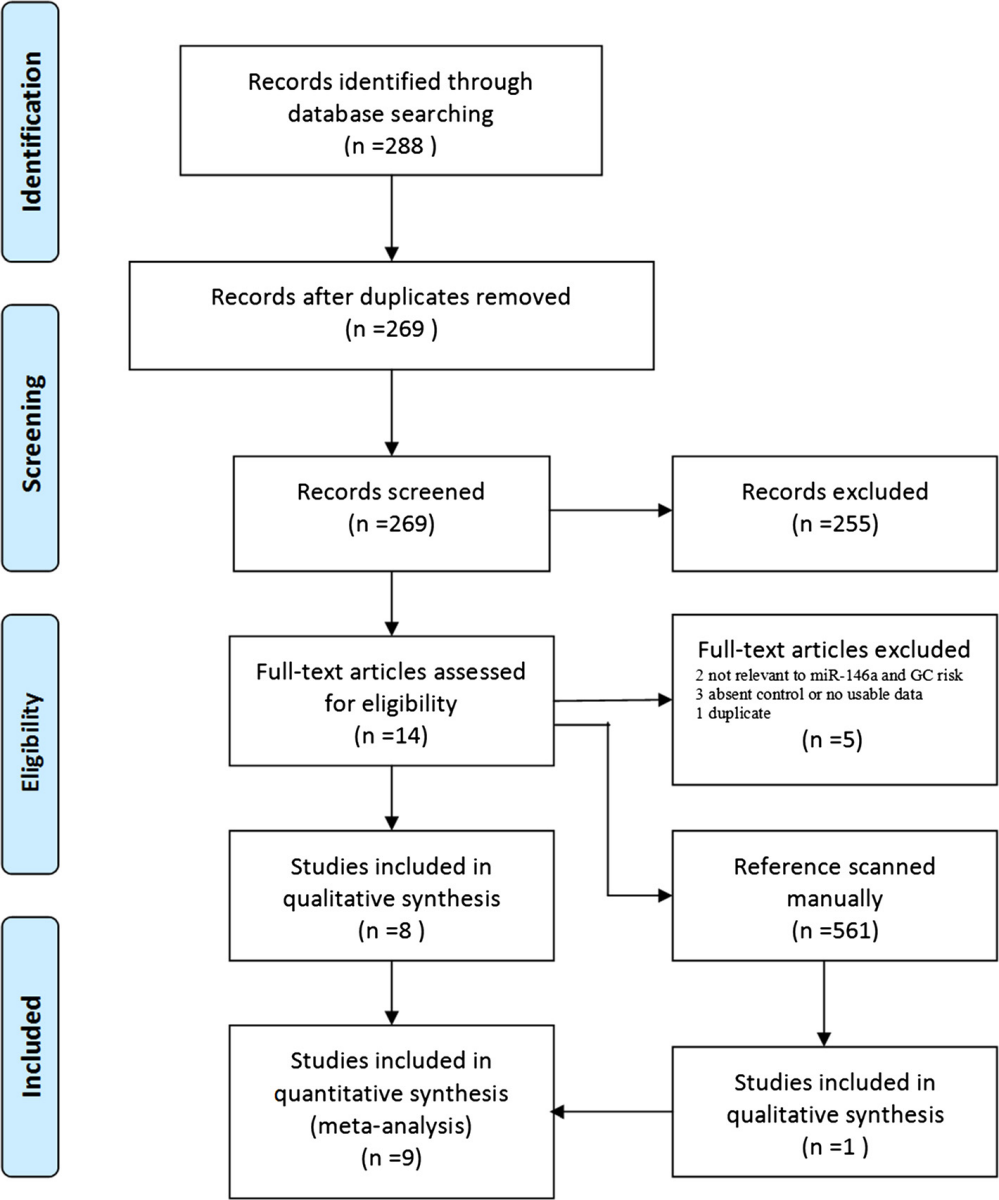
**Flow chart of Studies inclusion and exclusion.**

A total of 288 articles were identified from the PubMed, ISI and CBM database. The flow chart summarizes the literature review process as shown in Figure 1. A total of nine studies involving 3,885 GC cases and 5,396 controls were ultimately pooled for the present meta-analysis [23-31]. The characteristics of these studies are listed in Table 1. Among them, seven studies were performed in Asia (including patients from China, Japan and Korea), two studies in Europe (including patients from Greece, Germany, Lithuania and Latvia). Article by Okubo et al. [32] was excluded because of the duplication with another study [23]. All of the included articles were hospital-based case–control studies. The distribution of genotypes in the controls of all nine studies was in agreement with HWE.

**Table 1:** **Characteristics of studies included in the meta-analysis**

**First author**	**Year**	**Location**	**Genotyping method**	**No. of cases**	**No. of controls**	**Cases**			**Controls**			**P**_**HWE**_ **for control**
						**CC**	**CG**	**GG**	**CC**	**CG**	**GG**	
Okubo	2010	Japan	PCR-RFLP	552	697	236	243	73	254	322	121	0.2776
Zeng	2010	China	PCR-RFLP	304	304	89	153	62	119	132	53	0.1223
Hishida	2011	Japan	PCR-CTPP	583	1637	230	271	82	633	775	229	0.7381
Zhou F	2012	China	TaqMan MGB	1686	1895	286	822	578	393	951	551	0.6407
Zhou Y	2012	China	Locus-specific PCR	299	416	107	146	46	127	215	74	0.301
Ahn	2013	Korea	PCR-RFLP	461	447	159	231	71	164	221	62	0.3618
Dikeakos	2014	Greece	PCR-RFLP	163	480	105	45	13	307	149	24	0.2892
Kupcinskas	2014	Europe	Real Time-PCR	362	347	16	94	252	16	108	223	0.5311
Pu	2014	China	PCR-RFLP	197	513	65	96	36	143	274	96	0.0801

### Quantitative data synthesis

**Figure 2:**
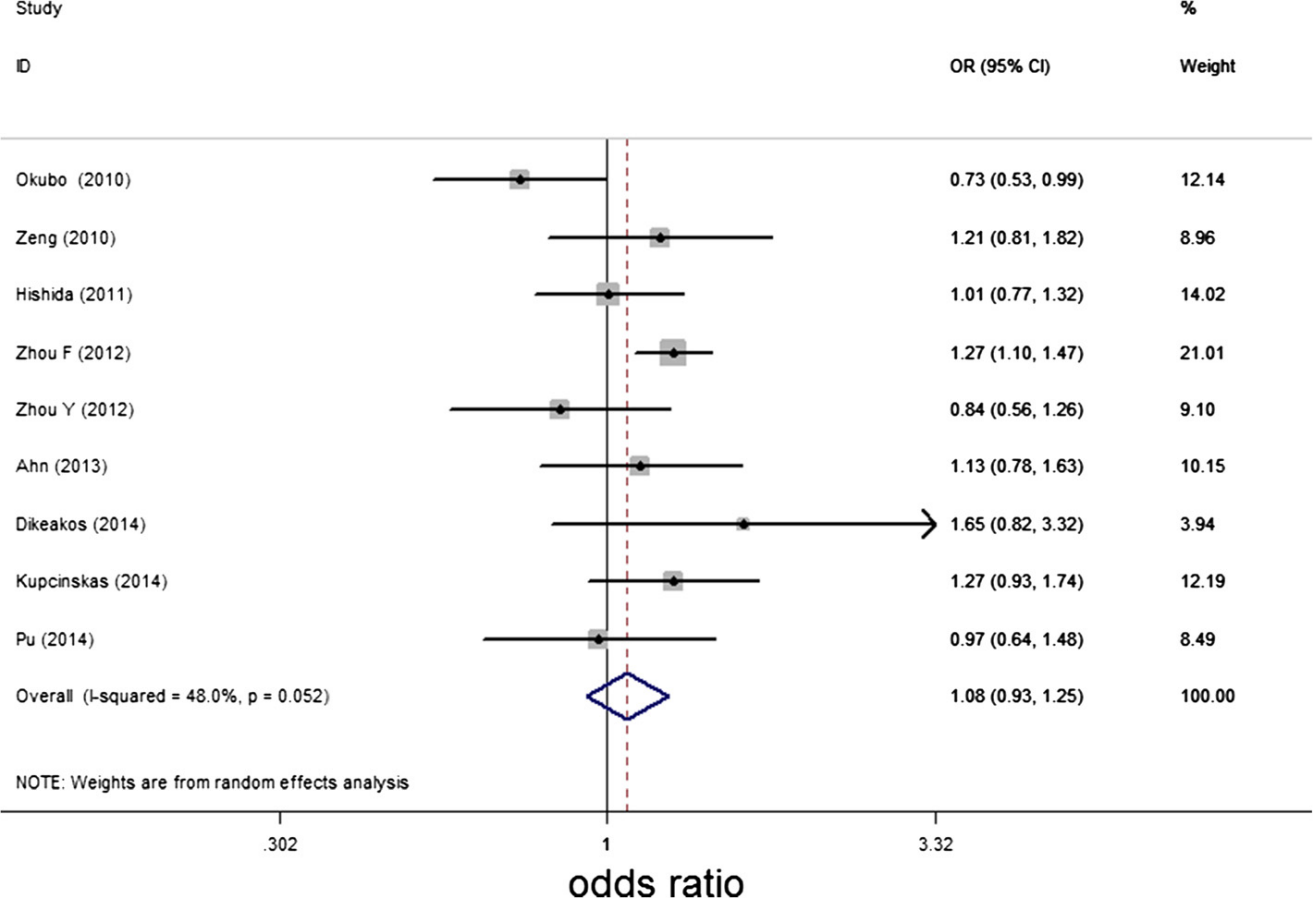
**Meta-analysis of miR-146a rs2910164 polymorphism and gastric cancer susceptibility for recessive model (GG vs. CG + CC).**

The summary of this meta-analysis for the association between miR-146a rs2910164 G/C polymorphism and the susceptibility of gastric cancer were shown in Table 2. Overall, there was no significant association in genotype distribution between gastric cancer and control by combining the nine studies. The random-effects model was used to pool these results as statistically heterogeneity was observed between studies (*P*_Q-test_ <0.1, *I*^2^ > 50%). The pooled OR estimates of the meta-analyses were often greater than 1.00, indicating a potential association of rs2910164 polymorphism and GC although this effect was not statistically significant. For recessive model, as *P*_Q-test_ <0.1 by heterogeneity analysis, also the random-effects model was used for analysis. As shown in Figure 2, there was no statistically increased risk of gastric cancer in recessive model when pooled the nine studies (recessive model, GG vs. CG/ CC: OR = 1.076, 95% CI 0.925-1.251, P = 0.342).

**Table 2:** **Meta-analysis of miR-146a rs2910164 polymorphism with gastric cancer by population**

**Population**	**Comparison**	**No. of study**	**Test of association**		**Test of heterogeneity**	
			**OR (95% CI)**	***P***	***P *** **(Q-test)**	***I*** ^**2**^
Asian	GG vs. CC	7	1.016(0.780, 1.324)	0.906	0.000	75.2%
	CG vs. CC		0.998(0.847, 1.176)	0.982	0.014	62.2%
	CG/GG vs. CC		1.005(0.830, 1.216)	0.962	0.001	75.1%
	GG vs. CG/CC		1.025(0.861, 1.221)	0.779	0.036	55.6%
	G vs. C		1.006(0.877, 1.153)	0.935	0.000	78.7%
European	GG vs. CC	2	1.331(0.802, 2.210)	0.269	0.512	0.0%
	CG vs. CC		0.880(0.619, 1.253)	0.478	0.973	0.0%
	CG/GG vs. CC		0.994(0.716, 1.380)	0.971	0.875	0.0%
	GG vs. CG/CC		1.326(0.995, 1.767)	0.054	0.512	0.0%
	G vs. C		1.146(0.937, 1.401)	0.184	0.608	0.0%
Overall	GG vs. CC	9	1.056(0.836, 1.334)	0.649	0.001	68.1%
	CG vs. CC		0.984(0.853, 1.136)	0.830	0.037	51.3%
	CG/GG vs. CC		1.005(0.852, 1.184)	0.956	0.002	66.9%
	GG vs. CG/CC		1.076(0.925, 1.251)	0.342	0.052	48.0%
	G vs. C		1.028(0.914, 1.155)	0.648	0.000	72.5%

**Figure 3:**
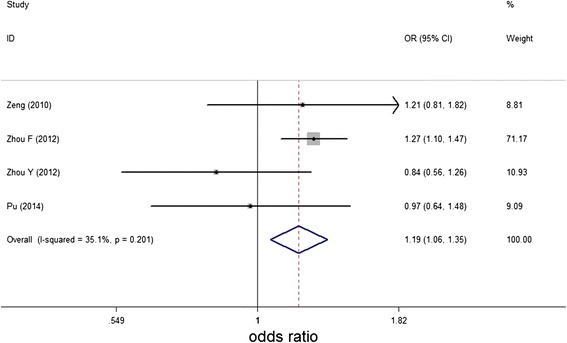
**Meta-analysis of miR-146a rs2910164 polymorphism and gastric cancer susceptibility in Chinese for recessive model (GG vs. CG + CC).**

However, as shown in Table 3 and Figure 3, when we performed subgroup analysis by location, the combined result showed that miR-146a rs2910164 GG genotype was associated with increased gastric cancer risk in China for recessive model (GG vs. CG/ CC: OR = 1.193, 95% CI 1.057-1.346, P = 0.004). In addition, interactions between miR-146a polymorphism and clinicopathological characters were also conducted when usable data obtained. No significant association between miR-146a genetic variant and gastric cancer risk were found in the stratified analysis by gender, smoking, location, metastasis of lymph node and Laurèn’s classification (Table 4).

**Table 3:** **Meta-analysis of miR-146a rs2910164 polymorphism with gastric cancer in Chinese**

**Comparison**	**Test of association**		**Test of heterogeneity**	
	**OR (95% CI)**	***P***	***P *** **(Q-test)**	***I*** ^**2**^
GG vs. CC	1.113(0.773, 1.604)	0.564	0.011	73.1%
CG vs. CC	1.045(0.783, 1.394)	0.766	0.012	72.8%
CG/GG vs. CC	1.065(0.779, 1.457)	0.692	0.002	79.4%
GG vs. CG/CC	1.193(1.057, 1.346)	0.004	0.201	35.1%
G vs. C	1.055(0.868, 1.283)	0.590	0.004	77.8%

**Table 4:** **Meta-analysis of rs2910164 polymorphism with GC by Clinicopathological characters**

**Parameters**	**Comparison**	**No. of study**	**Test of association**		**Test of heterogeneity**	
			**OR (95% CI)**	***P***	***P*** **(Q-test)**	***I*** ^**2**^
Gender	CC vs. GG/CG	4	0.916 (0.691, 1.215)	0.542	0.201	37.7%
(Male vs. Female)	CC/CG vs. GG	4	0.939 (0.767, 1.148)	0.538	0.967	0.0%
Smoking (No vs. Yes)	CC vs. CG/GG	2	0.843 (0.554, 1.282)	0.425	0.315	0.9%
Site (Cardic vs. Non-Cardic)	CC vs. CG/GG	4	0.844 (0.593, 1.201)	0.346	0.382	0.0%
	CC/CG vs. GG	4	0.897 (0.738, 1.090)	0.274	0.549	0.0%
Metastasis of lymph node (Negative vs. Positive)	CC vs. CG/GG	2	1.211(0.842, 1.742)	0.302	0.589	0.0%
Laurèn’s classification (Intestinal vs. Diffuse)	CC/CG vs. GG	4	0.961(0.807, 1.145)	0.658	0.655	0.0%

### Sensitivity analysis and publication bias

**Figure 4:**
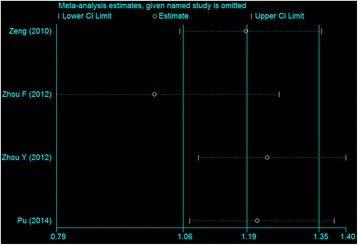
**The influence of each study by omission of individual studies in China for recessive model.**

We use one-way sensitivity analysis to evaluate the stability of the result in China [33]. In recessive model, the sensitivity analysis of pooled OR with 95%CI changed from 1.193(1.057, 1.346) to 0.995(0.786, 1.260) when omitting one study by Zhou F [26] (Figure 4). The corresponding pooled OR was not materially altered in other genetic models.

**Figure 5:**
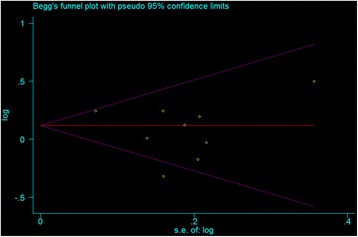
**Begg’s funnel plot with pseudo 95% confidence limits.**

**Figure 6:**
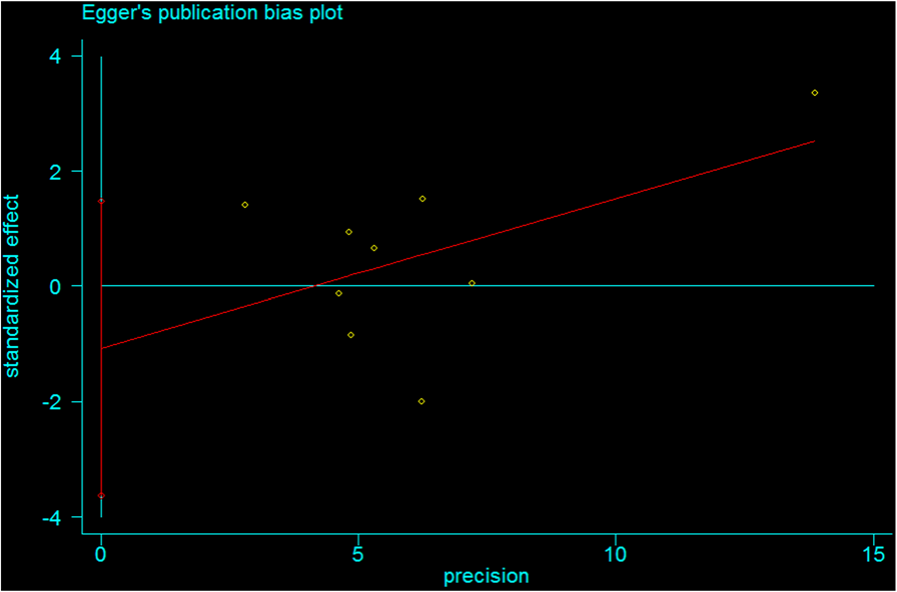
**Egger’s publication bias plot.**

Begg’s funnel plot (Figure 5) and Egger’s test (Figure 6) indicated no potential publication bias (*P* = 1.000 and 0.355, respectively). The funnel plots showed some asymmetry, which is probably due to the limited number of studies.

## Discussion

MiR-146a has been shown to play an important role in cell proliferation, differentiation, apoptosis and tumorigenesis [34-37]. Study by Jazdzewski et al. [38] in 2008 has suggested that rs2910164, a common polymorphism in the pre-miR-146a sequence, could alter mature miR-146a expression and play a role in the tumorigenesis. In the past three years, a number of meta-analyses and review pooled the association between rs2910164 G > C polymorphism and cancer risk including gastrointestinal tumors broadly [15-19,39-43]. Nine of them have evaluated the association between rs2910164 polymorphism and gastric cancer risk. With the same results, all of them concluded no significant association between rs2910164 polymorphism and gastric cancer.

Still why we performed this meta-analysis concerning the miR-146a polymorphism in gastric cancer susceptibility? Firstly, as shown in Table 5, the included studies were limited in all of these meta-analyses regarding gastric cancer. All of them excluded the study by Zhou Y et al. [27] because of absence data in the article. Among them, meta-analysis by Xu X et al. [19] included six researches [23-26,28,44] to evaluate the association between rs2910164 polymorphism and gastric cancer risk. However, one of the six included articles detected rs2910164 polymorphism from gastritis [44], not gastric cancer. Then, none of the nine meta-analyses evaluated the miR-146a rs2910164 G > C polymorphism and gastric cancer susceptibility in details. Moreover, it is general that esophageal cancer, liver cancer, gastric cancer, cervical cancer and other malignancies have too distinct molecular mechanisms and logically they do not seem to harbor a common polymorphism as a risk factor. Similarly, esophageal cancer, gastric cancer and colorectal cancer are not always consistent with each other in this term. Last but not least, an update is needed to evaluate the association between rs2910164 G > C polymorphism and gastric cancer risk. Therefore, we try to acquire a more precise assessment of the association between rs2910164 polymorphism and gastric risk.

**Table 5:** **Meta-analyses of rs2910164 polymorphism and cancer risk (information about GC)**

**First author**	**Year**	**Included study about gastric cancer**					
		**[** 24 **]**	**[** 25 **]**	**[** 26 **]**	**[** 28 **]**	**[** 23 **]**	**[** 44 **]**
Srivastava [15]	2012	√	√	√		√	
Wang J [16]	2012	√	√			√	
Wang F [17]	2012	√	√			√	
Wu [18]	2013	√	√			√	
Xu X [19]	2014	√	√	√	√	√	√
Qiu [40]	2011	√	√			√	
He [41]	2012	√	√	√		√	
Xu Y [42]	2013	√	√	√		√	
Yin [43]	2013	√	√	√		√	

Focused on rs2910164 SNP and gastric cancer, the present meta-analysis showed that the miR-146a GG genotype was associated with increased risk of gastric cancer in China subgroup in recessive model (pooled OR = 1.193). There were no significant differences in gender, smoking, location, metastasis of lymph node and Laurèn’s classification across genotypes.

Of the nine studies included, studies by Zeng et al. [24] and Zhou F et al. [26] describe distribution of genotype in gastric cancer by age. The former demonstrated that rs2910164 GC/GG genotype had a increased risk of gastric cancer especially in younger individuals aged (≤58 years), the later showed that GG genotype increased gastric cancer risk was more evident in younger subjects (≤65 years). Zhou Y et al. [27] did not find any differennt distribution in gastric cancer between different tumor size (<5 cm vs. ≥5 cm). In addition, Zeng et al. [24] reported no statistically significant association in the variant genotypes with tumor differentiation. Almost all of included studies were conducted in Asia except two studies were in Europe. The study by Kupcinskas et al. in Europe [30] analyzed gene polymorphisms of miR-146a in 995 subjects including Germany, Latvia and Lithuania. Data analysis showed that gene polymorphisms of miR-146a are not associated with risk of GC in Europe. Besides, no difference between diffuse and intestinal-type gastric cancer groups was found.

The results should be interpreted with carefulness because of several limitations. First, the limited patient numbers may have influence the outcomes. Although we conducted comprehensive literature research from multiple databases with no restriction in publication languages, dates, ethnicities and other factors, there were only a total of nine studies involving 3,885 gastric cancer cases and 5,396 controls for the present meta-analysis. The overall OR indicated a potential association between rs2910164 polymorphism and GC but the effect was not statistically significant. It is still hard to make a firm conclusion about the accuracy of association between miR-146a rs2910164 G > C polymorphism and gastric cancer susceptibility. Second, we use one-way sensitivity analysis to evaluate the stability of the meta-analysis and the result altered when omitting the study with the largest samples. Third, only a few included researches reported data of interactions between miR-146a polymorphism and clinicopathological characters in gastric cancer, and all of the pooled analysis of interactions was limited by small sample size and deficit of data.

## Conclusion

In conclusion, the present meta-analysis suggests an increased risk between miR-146a rs2910164 GG genotype and gastric cancer susceptibility in Chinese based on published literatures. Further studies on a large scale may be needed to confirm the genetic susceptibility of miR-146a rs2910164 in gastric cancer.
